# The Effort, Dyspnea, and Cooperation Scores in Mild and Moderate Post-COVID-19 Patients: Results of a Retrospective Study

**DOI:** 10.3390/arm93050043

**Published:** 2025-10-07

**Authors:** Ovidiu Cristian Chiriac, Corina Sporea, Daniela Miricescu, Ana Raluca Mitrea, Ileana Adela Vacaroiu, Raluca Grigore, Adriana Sarah Nica

**Affiliations:** 1 Discipline of Balneophysiokinetotherapy and Recovery, Faculty of Midwifery and Nursing, Carol Davila University of Medicine and Pharmacy, 050474 Bucharest, Romania; ovidiu-cristian.chiriac@drd.umfcd.ro; 2 Department of Rehabilitation, Physical and Balneotherapy Medicine I, Central Military Emergency University Hospital “Dr. Carol Davila”, 010825 Bucharest, Romania; 3 Scientific Research Core, National University Center for Children’s Neurorehabilitation “Robănescu-Pădure”, 44 Dumitru Minca Street, 041408 Bucharest, Romania; 4 Discipline of Biochemistry, Faculty of Dentistry, Carol Davila University of Medicine and Pharmacy, 8 Eroii Sanitari Blvd., 050474 Bucharest, Romania; 5 Physical Medicine and Rehabilitation (Medical Recovery Neurology), Carol Davila University of Medicine and Pharmacy, 050474 Bucharest, Romania; ana-raluca.vacaroiu@rez.umfcd.ro; 6 Department of Nephrology, Faculty of Medicine, Carol Davila University of Medicine and Pharmacy, 020021 Bucharest, Romania; ileana.vacaroiu@umfcd.ro; 7 ENT, Head & Neck Surgery Department, Coltea Clinical Hospital, Carol Davila University of Medicine and Pharmacy, 022328 Bucharest, Romania; raluca.grigore@umfcd.ro; 8 Department of Physical Medicine and Rehabilitation, Carol Davila University of Medicine and Pharmacy, 050474 Bucharest, Romania; sarah.nica@umfcd.ro

**Keywords:** isolation, muscle weakness, effort, dyspnea, recovery, physical exercises

## Abstract

**Highlights:**

**What are the main findings?**

- Post-COVID-19 patients with mild and moderate forms of the disease showed significant improvements in cooperation, effort, and dyspnea scores following a structured rehabilitation program.
- Improvements were observed across all demographic subgroups (by gender and age), although some variation in magnitude was noted.

**What is the implication of the main finding?**

- These results highlight the effectiveness of personalized rehabilitation in enhancing not only physical capacity but also patient engagement and respiratory comfort during post-COVID-19 recovery.
- The identified correlations and demographic patterns may guide targeted interventions and resource allocation, supporting more individualized and symptom-oriented rehabilitation strategies.

**Abstract:**

COVID-19 signs and symptoms varied among patients, with the most common being fever, fatigue, sore throat, cough, anorexia, and shortness of breath. (1) Background: This study aimed to assess effort, dyspnea, and cooperation scores in patients with mild and moderate post-COVID-19 forms, both at baseline and after completing a structured physical recovery program. (2) Methods: Our study included 160 post-COVID-19 patients who had experienced mild or moderate disease. (3) Results: Effort and dyspnea scores were significantly lower (*p* < 0.01), while cooperation scores were significantly higher after the rehabilitation program. Both men and women demonstrated significant increases in cooperation scores after recovery. Additionally, both groups showed statistically significant reductions in effort and dyspnea scores (*p* < 0.001). Among patients aged under and over 60 years, effort and dyspnea scores decreased after rehabilitation, and cooperation scores increased significantly (*p* < 0.001). No statistically significant differences were observed between genders in any of the three scores. Similarly, no significant differences by age were found in cooperation or dyspnea scores. A significant negative correlation was observed between cooperation and effort scores: patients with higher cooperation scores tended to report lower effort scores, and vice versa (*p* < 0.001, R = −0.571). (4) Conclusions: The improved cooperation demonstrated by patients during the physical recovery program was significantly associated with reductions in perceived effort and dyspnea, indicating a positive impact on post-COVID-19 rehabilitation outcomes.

## 1. Introduction

Severe Acute Respiratory Syndrome Coronavirus 2 (SARS-CoV-2) affected people worldwide, leading to increased morbidity and mortality [1]. COVID-19 signs and symptoms varied among patients, with fever, fatigue, expectoration, cough, anorexia, and shortness of breath being the most common [2]. In some cases, symptoms such as sore throat, confusion, headache, nausea, diarrhea, and gastrointestinal issues were also reported [2]. Moreover, among older patients, COVID-19 infection was linked to “brain fog” or could cause respiratory complications [3]. Moderate forms of COVID-19 infection were associated with various symptoms such as sore throat, fever, dry cough, nausea, vomiting, abdominal pain, and loose stools [4,5]. Symptoms similar to pneumonia, such as a fever and cough without signs of hypoxemia, along with notable lesions observed on chest CT, were also reported in patients with moderate disease [5].

Dyspnea is defined as “a subjective experience of breathing discomfort that consists of qualitatively distinct sensations that vary in intensity”. Work or effort to breathe, difficulty with inspiration or air hunger, and a tight chest are three well-studied features of dyspnea that involve different neurological pathways [6]. Typically, dyspnea is a primary symptom in patients with chronic lung disease and is commonly used to assess chronic obstructive pulmonary disease (COPD). The Shortness of Breath Questionnaire (SOBQ), Borg Scale (Borg), and Visual Analog Scale (VAS) are standard tools for dyspnea assessment [7,8].

Fatigue results from multiple factors, affecting both peripheral muscle contractile function and central mechanisms related to the descending drive to active muscles. SARS-CoV-2 infection impairs oxygen delivery to skeletal muscles, likely influencing fatigue levels [9]. Therefore, Borg’s Rating of Perceived Exertion (RPE) is a widely used psychological tool to assess the subjective perception of effort during exercise [10].

The level of patient cooperation relates to their participation, responsiveness, and engagement with therapeutic advice—an essential yet often overlooked aspect of rehabilitation research. Factors such as physical symptoms, mental health, trust in the care team, social support, and prior healthcare experiences can all affect cooperation [11].

In post-COVID-19 recovery scenarios, where patients may still experience fatigue, anxiety, and uncertainty, cooperation becomes even more vital for following rehab plans and achieving successful outcomes [12,13,14].

Furthermore, the connection between subjective effort, dyspnea scores, and the patient’s level of cooperation remains poorly documented, despite being intuitively relevant. A better understanding of this relationship could help clinicians predict which patients are more likely to actively engage in therapy and which may require additional motivation or psychological support. Similarly, recognizing patterns of progress or stagnation across different age and sex groups can guide resource allocation, patient education, and the development of tailored treatment plans.

In this context, the present retrospective study aimed to analyze how cooperation, effort, and dyspnea scores evolved in patients recovering from mild and moderate COVID-19. Conducted within a structured rehabilitation setting, the study included detailed functional assessments during both the initial phase and after recovery. The main goals were to: (1) compare the initial and post-recovery scores for the three measures; (2) evaluate how gender and age affect score changes; and (3) explore potential correlations between effort, dyspnea, and cooperation scores.

By addressing these objectives, our study aims to enhance current understanding of post-COVID-19 rehabilitation by emphasizing subjective yet clinically relevant indicators. It highlights the importance of incorporating the patient’s lived experience—how they feel, breathe, and respond behaviorally—into the overall recovery process. Additionally, it supports the idea that rehabilitation should not be guided solely by objective measures such as lung function tests or imaging results but should also include qualitative aspects that reflect patient engagement and perceived well-being.

By emphasizing the complex relationship between physiological recovery, symptom perception, and therapeutic cooperation, this study supports broader efforts to develop more holistic, patient-centered rehabilitation methods. It also highlights the importance of tailoring rehabilitation protocols not only to disease severity but also to individual factors like age, sex, and motivation levels.

A deeper understanding of these factors may ultimately enhance rehabilitation services, result in improved patient outcomes, and help develop adaptable, evidence-based care pathways for individuals recovering from COVID-19 and other complex health conditions.

## 2. Materials and Methods

This retrospective study involved 160 patients with mild or moderate post-COVID-19 infection who underwent two assessment phases. The first evaluation took place after their initial infection during hospitalization. The patients started their physical exercises in the hospital and continued the recommended exercises at home. The home recovery program lasted at least two weeks, and they were reevaluated after a subsequent hospital visit.

### 2.1. Clarification of Disease Severity Classification

In accordance with internationally recognized guidelines, the current study uses the terms mild and moderate-to-severe to classify COVID-19 severity. The mild category refers to patients who experienced symptoms such as fever, fatigue, cough, or anosmia, but without shortness of breath, dyspnea, or abnormal chest imaging, as defined by the National Institutes of Health (NIH). The moderate-to-severe group includes individuals who exhibited signs of respiratory involvement (e.g., dyspnea, reduced oxygen saturation below 94%) or required supportive medical care. This classification is consistent with both NIH and World Health Organization (WHO) recommendations [15].

The study was approved by the hospital’s Ethical Committee (Approval No. 694/03/28/2024), and all procedures were conducted in accordance with the ethical standards outlined in the Declaration of Helsinki.

### 2.2. Functional Evaluation Protocol

Functional assessment was conducted according to the International Classification of Functioning, Disability and Health (ICF) developed by the World Health Organization. The assessment included three main components:

- Effort was measured using the Borg Rating of Perceived Exertion (RPE) Scale [16], which allows patients subjectively rate how hard they feel they are working on a scale from 0 to 10:

0—No exertion;

0.5—Very, very light;

1—Very light;

2—Light;

3—Moderate;

4—Somewhat hard;

5—Hard;

7—Very hard;

9—Very, very hard;

10—Maximal exertion.

This scale was administered under standardized conditions during physical rehab sessions, both at baseline and follow-up, to assess patients’ perceived physical effort.

Perceived effort and dyspnea were both evaluated using the Modified Borg Scale (0–10), administered immediately after standardized physical therapy tasks such as transfers, walking along the corridor, or therapeutic exercises, which were individually adapted to the patient’s physical abilities. This approach ensured that both perceived exertion and breathlessness were assessed under exertional conditions, providing a consistent and clinically relevant snapshot of functional tolerance.

- Dyspnea was assessed using the Modified Borg Dyspnea Scale [17], a validated instrument for rating perceived breathlessness. Patients were asked to rate their sensation of dyspnea on a scale from 0 to 10, with descriptive anchors as follows:

0—No dyspnea;

1—Very slight;

2—Slight;

3—Moderate;

4—Somewhat severe;

5—Severe;

6—More severe;

7—Very severe;

8—Extremely severe;

9—Almost maximal;

10—Maximal breathlessness.

The scale measures the subjective perception of respiratory effort during physical activity and was administered under standardized conditions during both initial and follow-up assessments.

In addition, dyspnea was evaluated during the same rehabilitation tasks—such as walking, transfers, or moderate exercise—rather than at rest. Patients were instructed to indicate their perceived breathlessness immediately after completing the activity, ensuring a consistent and clinically relevant measurement of exertional dyspnea.

- Cooperation was assessed using the Score Five Questions (S5Q) scale [18,19], which measures how well patients cooperate during the acute and subacute stages of illness. The scale includes five simple commands, each scored as 0 (no response) or 1 (correct response).
  (a) Open and close your eyes!
  (b) Look at me!
  (c) Open your mouth and stick out your tongue!
  (d) Nod your head up and down!
  (e) Raise your eyebrows and hold for a count of five.

The S5Q scale provides an objective assessment of the patient’s capacity to follow basic verbal instructions, which is crucial for starting an active rehabilitation program.

Scores were recorded at two time points: (1) the initial assessment, conducted upon admission to the recovery ward, and (2) a follow-up assessment, conducted during the subsequent hospitalization.

### 2.3. Rehabilitation Protocol

During hospitalization, all patients participated in a rehabilitation program and continued their exercises at home. Physiotherapists, Kinesio therapists, and rehabilitation physicians supported patients with their rehabilitation plans. The recovery schedule was individualized for each patient based on their functional level and included daily sessions consisting of:

- Respiratory physiotherapy including breathing retraining, postural correction to enhance bronchial drainage, and incentive spirometry.
- Joint mobilization to preserve mobility and prevent deconditioning.
- Muscle strengthening and endurance training for both upper and lower limbs.
- Balance and coordination exercises, such as sitting, standing, and walking retraining.
- Occupational therapy adapted to each patient’s level of independence and cognitive status.

Respiratory rehabilitation played a key role, regardless of functional status. This included diaphragmatic breathing exercises performed in a supine position with knees bent, posture improvement, bronchial drainage techniques, and low-intensity aerobic exercises to enhance cardiorespiratory endurance. Patients were encouragedto change positions every two hours during the day (supine, lateral, prone).

Standardized daily routines included upper and lower limb mobility exercises performed at a controlled pace with 10 repetitions for each movement, along with gradual verticalization assisted by staff to prevent balance issues.

After discharge, each patient was given a personalized home exercise program that replicated techniques practiced in the hospital. They were instructed to continue these exercises on their own until the next scheduled hospitalization, when a functional reassessment was performed.

### 2.4. Statistical Analysis

IBM SPSS Statistics 27 was used to analyze the results. Figures and tables were created using Microsoft Excel and Word 2024. Quantitative variables are presented as means with standard deviations or medians with interquartile ranges (IQR). The Shapiro–Wilk test was used to assess the normality of the distributions TaT [20].

For within-subject comparisons between initial and post-recovery values (paired samples), the Wilcoxon Signed Rank Test was applied, as most variables did not follow a parametric distribution [21]. For between-group comparisons (e.g., gender, age subgroups), the Mann–Whitney U test was employed [22]. Correlations between non-parametric variables, such as effort and dyspnea scores, were examined using Spearman’s rho correlation coefficient.

The threshold for statistical significance was set at α = 0.05.

Although the retrospective design and absence of a formal control group may limit causal interpretations, the internal comparison of pre- and post-rehabilitation scores provides valuable insight into short-term functional dynamics during post-COVID-19 recovery. The study’s pragmatic design reflects real-world clinical scenarios in a high-volume rehabilitation center.

## 3. Results

The cohort included a total of 160 adult patients previously diagnosed with mild or moderate forms of COVID-19. Of these, 97 were women, accounting for 60.6% of the study population. The overall mean age was 58.54 years, with a standard deviation of 13.3 years. The median age was 58, and the interquartile range (IQR) ranged from 47 to 70 years. This age distribution highlights a predominance of individuals in the late middle-aged to elderly category. Nearly half of the participants (46.5%) were aged 60 years or older, reflecting the increased vulnerability of this age group to both the functional impairments caused by COVID-19 and the need for post-acute rehabilitation. The demographic structure of the cohort is representative of the broader population requiring multidisciplinary recovery strategies following COVID-19 infection.

### 3.1. Descriptive and Comparative Analysis of Subjective Scores

Table 1 presents the comparative values of cooperation, perceived effort, and dyspnea scores recorded at the initial assessment and after completion of the recovery program, for the overall cohort, as well as separately for women and men. The data include mean ± standard deviation and median (interquartile range), along with the results of statistical significance testing. All three parameters showed statistically significant intra-individual improvements following rehabilitation, regardless of sex.

### 3.2. Age-Stratified Comparison of Subjective Recovery Outcomes

Table 2 displays the initial and post-recovery scores for cooperation, effort, and dyspnea, stratified by age groups (<60 years and ≥60 years). While both subgroups demonstrated statistically significant improvements across all three parameters, patients under 60 years reported slightly greater absolute improvements. These findings suggest consistent benefits of the rehabilitation program across age categories, with modest age-related variations in recovery dynamics.

### 3.3. Combined Analysis by Gender and Age

Table 3 details the pre- and post-recovery scores for cooperation, effort, and dyspnea, simultaneously stratified by gender and age groups. This integrative approach aims to identify potential interactions between these demographic factors in shaping short-term rehabilitation outcomes. Despite some variability in the magnitude of improvements across subgroups, no statistically significant interaction effects were observed, supporting the overall effectiveness of the intervention across different patient profiles.

### 3.4. Correlations Between Baseline Subjective Scores

**Figure 1:**
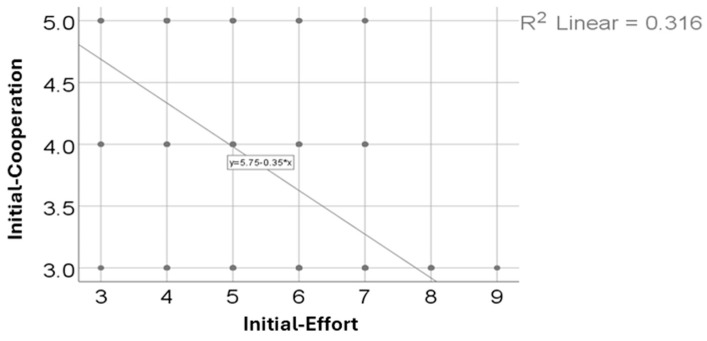
Correlation between initial cooperation and effort scores.

The association between initial cooperation and effort scores was assessed using Spearman’s rho correlation. As shown in Table 4 and Figure 1, a statistically significant inverse correlation was identified (R = –0.571, *p* < 0.001). This suggests that patients who were more cooperative at baseline tended to report lower levels of perceived effort during the initial rehabilitation stage.

**Figure 2:**
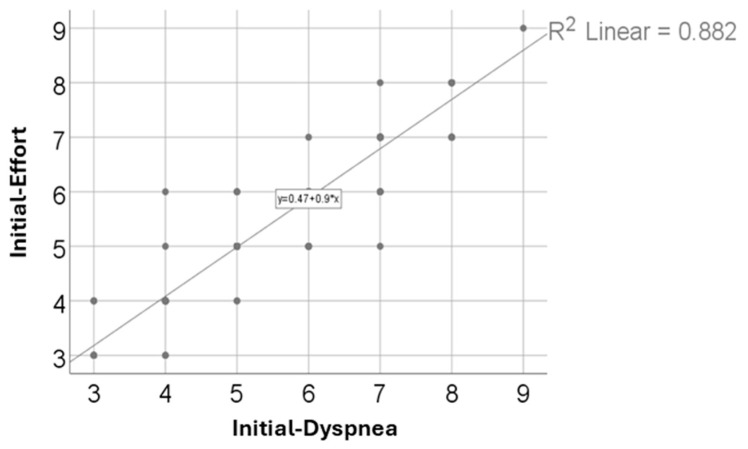
Correlation between initial effort and dyspnea scores.

Similarly, Table 5 and Figure 2 show a very strong positive correlation between initial effort and dyspnea scores (R = 0.942, *p* < 0.001), indicating that patients who experienced greater exertion also tended to report more severe dyspnea. This linear relationship was statistically robust.

## 4. Discussion

Depending on the severity, COVID-19 infection has been associated with reduced exercise tolerance and capacity, fatigue, neurocognitive problems, muscle pain, and shortness of breath [23]. Physical exercise has been used to help rehabilitate patients with cardiopulmonary complications, cancer, or cancer therapy-related issues, as well as mental health disorders [24].

Due to social isolation and persistent concerns about infection, mental health disorders related to the COVID-19 crisis remain significant [25]. Sedentary behaviors, reduced physical activity, and poor dietary habits have been linked to negative mental and physical health outcomes, including a higher risk of depression, anxiety, psychological distress, and weight gain [26].

Research shows that symptoms of depression and anxiety increased during the pandemic, with certain groups, such as women and people living in areas with high COVID-19 incidence, being at greater risk of mental health deterioration [27,28]. The study by Alacevich et al. examined the co-occurrence of physical and mental health conditions during and after symptomatic SARS-CoV-2 infection. It confirmed that physical symptoms such as anosmia, high fever, persistent cough, and breathlessness, were significantly associated with moderate and severe anxiety and depression, both during COVID-19 the illness and after recovery [29]. Furthermore, most healthcare professionals experienced significant anxiety, especially during the pandemic [30]. Observational studies suggest that encouraging physical activity and reducing sedentary behavior can help protect mental health in children and adolescents [31]. Among students, low-to-moderate-intensity aerobic exercises interventions appear most effective in improving mental health—reducing depressive symptoms and perceived stress—within just a few weeks. Additionally, short-term sessions of certain types of exercises, especially yoga, appear to be particularly effective in immediately altering perceptions of bodily signals, cardiac activity, and emotion processing [32]. Silva LAD reported that the aquatic exercise reduces depression and anxiety, improves functional independence, and decreases oxidative stress in elderly persons with depression [33]. A study conducted by Cunningham found that physical activity is strongly associated with life satisfaction and happiness among young, middle-aged, and older adults, with both indicators tending to increase with age [34].

Typically, patients experiencing persistent post-COVID-19 symptoms are advised to follow a home-based respiratory muscle training program, often supervised through telerehabilitation, which improves their quality of life [35].

The current study aimed to examine changes in perceived effort, dyspnea, and cooperation among mild and moderate post-COVID-19 patients undergoing structured rehabilitation, using a repeated-measurement design that enabled intra-individual comparisons between initial assessment and post-recovery. These parameters, although subjective, reflect important aspects of patient functionality and interaction during the subacute and recovery phases of the disease.

### 4.1. Overall Improvements in Effort, Dyspnea, and Cooperation

The findings showed statistically significant improvements in all three areas after the rehabilitation period. Effort scores decreased considerably, indicating less perceived exertion during standardized tasks, which aligns with ongoing cardiovascular and muscular recovery.

The administration of the Modified Borg Scale during structured therapeutic activities, such as assisted walking or transfer exercises, enabled consistent tracking of perceived physical exertion across sessions. While inherently subjective, this tool proved valuable in the absence of objective benchmarks, especially given the variability in clinical status and exercise tolerance among post-COVID-19 patients. Its sensitivity to intra-individual change rendered it particularly useful for monitoring short-term rehabilitation progress.

Dyspnea scores followed a similar trend, suggesting a reduction in respiratory distress, which is often prolonged in patients with moderate and severe forms of COVID-19. Additionally, cooperation scores increased notably, likely reflecting both improved physical capacity and greater psychological readiness to participate in therapy programs (Table 1). Notably, dyspnea was assessed as exertional breathlessness, not at rest, since the Modified Borg Scale was administered immediately after structured rehabilitation activities. This method allowed for consistent comparisons of respiratory tolerance during effort-based tasks. Individual data points from these assessments are displayed in the correlation scatter plots (Figure 1 and Figure 2), highlighting the distribution of patient responses. The observed improvements likely reflect enhanced ventilatory efficiency and reduced respiratory discomfort during moderate physical effort. Furthermore, the narrowing of the score variability and the reduced number of outliers after recovery support the conclusion of functional respiratory improvement.

While the subjective improvements reported by patients are encouraging, the lack of objective functional or pulmonary assessments limits the interpretability of these findings from a physiological standpoint.

Although the absolute changes in perceived scores may appear modest (with average reductions of approximately 1 point on a 10-point scale), their statistical significance and consistent intra-individual patterns across the cohort suggest clinically meaningful progress. In rehabilitation settings, even small improvements in effort and dyspnea can translate into better tolerance for daily activities and increased adherence to therapy, particularly in the early stages of post-COVID-19 recovery. Therefore, while the improvements could be considered minimal in magnitude, their functional relevance should not be underestimated.

### 4.2. Gender-Based Analysis—Subtle Differences Without Significance

When analyzing gender-based changes, both male and female groups showed statistically significant improvements in effort, dyspnea, and cooperation individually (Table 1). However, none of the between-group comparisons were statistically significant, although men tended to have slightly greater gains, especially in cooperation scores. The lack of significant differences may be due to sample size variability or suggests that gender is not a major factor influencing short-term recovery outcomes in this context (Table 3).

Sex-stratified analyses were nonetheless retained to explore potential trends, considering previous reports of sex-related differences in physical performance, symptom perception, and recovery patterns. In this cohort, the similar trajectories observed in men and women support a generally uniform short-term response to rehabilitation across genders.

### 4.3. Age-Related Analysis—Consistent Improvements Across Age Groups

Comparative analysis by age (<60 vs. ≥60 years) also showed no statistically significant differences in the magnitude of improvement (Table 2). However, patients under 60 experienced slightly greater absolute gains in effort and dyspnea reduction. This pattern is expected given the physiological reserve and faster recovery typically observed in younger individuals, which was also associated with increased cooperation scores. Still, the fact that patients aged ≥ 60 also showed strong improvements supports the effectiveness of customized rehabilitation even in older populations, a group especially vulnerable to long-COVID effects. Comparison of differences in cooperation, effort, and dyspnea scores based on age did not reveal statistical significance (Table 3).

### 4.4. Strong Correlation Between Effort and Dyspnea

A particularly notable finding was the very strong positive correlation between initial effort and dyspnea scores (R = 0.942, *p* < 0.001), indicating a close link between these two perceived aspects. Patients who reported higher exertion also tended to experience more breathlessness, which can be explained both physiologically (for example, decreased oxygen uptake or ventilatory reserve) and behaviorally (such as deconditioning or anxiety). This relationship underscores the importance of integrated therapeutic approaches that address both components simultaneously rather than separately (Table 5).

### 4.5. Relationship Between Cooperation and Perceived Effort

A notable inverse correlation was found between initial cooperation and effort scores, indicating that patients who were more cooperative at baseline generally reported feeling less exertion during the early stages of recovery. Although the correlation was moderate in strength (Spearman’s rho, R = –0.571, *p* < 0.001), its statistical significance and consistency across the non-parametric distributions of both variables (Shapiro–Wilk, *p* < 0.05) underline a relevant behavioral dynamic in post-COVID-19 rehabilitation. This finding may reflect that individuals who are cognitively and motivationally more engaged—thus scoring higher on cooperation—are also better able to manage physical demands, either by optimizing movement strategies or by demonstrating greater psychological resilience in the face of exertional discomfort (Table 4 and Figure 1). These data align with existing literature emphasizing the importance of patient engagement in recovery trajectories [36] and support the idea that initial cooperative capacity may serve as a functional predictor for effort tolerance during rehabilitation. Although the R^2^ value in the linear model was relatively low—indicating modest variance explained—the Spearman correlation remained statistically robust. This apparent discrepancy highlights the importance of using non-parametric methods when analyzing ordinal clinical data. Clinically, the inverse association points to a potentially protective behavioral profile: individuals more engaged at baseline may experience lower perceived fatigue and show greater readiness for structured intervention. Overall, these findings reinforce the idea that early engagement and a positive therapeutic attitude may buffer the burden of physical exertion during subacute recovery.

### 4.6. Clinical Implications and Relevance to Rehabilitation Practice

These findings highlight the usefulness of subjective clinical scales for effort, dyspnea, and cooperation in tracking rehabilitation progress in post-COVID-19 patients. While objective measures (e.g., 6 min walk test, spirometry) remain crucial, the patient’s subjective experience offers vital information about functional limitations and engagement capacity. Improvements in cooperation particularly indicate not only better tolerance of physical demands but also improved emotional well-being and adherence to therapy.

### 4.7. Alignment with Existing Literature and Pathophysiological Considerations

The results align with previous reports on post-COVID recovery, which highlight ongoing fatigue, shortness of breath, and low functional compliance among patients weeks or even months after infection [14,37,38]. Research on cooperation, effort, and dyspnea scores in mild and moderate post-COVID-19 patients is limited. These assessments are usually conducted in patients with severe cases [39,40,41,42]. The post-COVID-19 rehabilitation program has been reported to improve dyspnea, anxiety, and kinesiophobia. Although pulmonary function results varied, improvements were seen in muscle strength, walking ability, sit-to-stand performance, and quality of life [43]. The study by Filipovic T et al. included 147 patients divided into two groups based on disease severity: the “mild to moderate group” (MMG) and the “severe stable group” (SSG). All patients underwent rehabilitation that included breathing exercises, range of motion exercises, and strength training, with intensity and progression tailored to each individual’s capacity. The study found significant improvements in all measured outcomes after the recovery program in both groups. When comparing the two groups, notable differences were found in the Borg scale for dyspnea and other measures [44]. Mild to moderate COVID-19 telerehabilitation has been shown to reduce dyspnea, improve performance, and enhance quality of life [45].

Pouliopoulou DV et al. analyzed various studies on the effects of rehabilitation programs, focusing on functional exercise capacity, muscle function, dyspnea, respiratory function, and quality of life in post-COVID-19 patients. The studies showed that rehabilitation programs were associated with improvements in functional exercise capacity, dyspnea, and quality of life [46].

Similar results were obtained by Elyazed TIA and coworkers, who investigated 68 post-COVID-19 patients complaining of fatigue, dyspnea, and exercise intolerance regarding the effects of a home-based pulmonary rehabilitation program. The rehabilitation group showed a significant decrease in fatigue and a significant improvement in dyspnea among patients [47].

Furthermore, Pleguezuelos et al. demonstrated that different exercise programs positively influence mental health, physical condition, immune system, and oxidative stress biomarkers in older patients with post-COVID-19 sequelae [48].

Our study provides additional detail by analyzing score changes within a structured rehabilitation setting, capturing both physical and psychological adaptation over time. The observed improvements may arise from multiple mechanisms, including the gradual reduction in pulmonary inflammation, improved neuromuscular control, and the psychosocial benefits of guided therapy.

### 4.8. On the Use of Subjective Assessment Tools

The use of subjective instruments such as the Borg and S5Q scales, while lacking objective psychological metrics, allowed for rapid and consistent evaluation across a large cohort, especially when objective measures were not feasible due to variable clinical severity and hospitalization duration. These tools are widely recognized in rehabilitation settings for capturing patient-reported outcomes.

The cooperation scale (S5Q), despite its apparent simplicity, proved clinically useful in identifying patients who were cognitively and behaviorally able to participate in structured rehabilitation. While it does not account for psychosocial variables, it served as a practical screening tool in the acute/subacute recovery phase.

The follow-up period was limited to the duration between two hospitalizations, which may not reflect long-term recovery. However, the significant short-term improvements observed after a single rehabilitation cycle underscore the importance of early intervention during the post-COVID-19 trajectory. Although the observed intra-individual improvements were statistically significant, the retrospective design and absence of a control group limit causal inference. These results should therefore be interpreted as associations rather than proof of efficacy. Future prospective studies are needed to determine the true effectiveness of this rehabilitation protocol.

Nevertheless, several limitations must be acknowledged to contextualize the findings and guide future work.

On the other hand, patients who survive severe COVID-19 develop a wide range of functional deficits, such as cardiopulmonary symptoms, neurological issues, and cognitive impairments [49]. Therefore, post-acute sequelae of COVID-19 affect multiple organ systems and cause various symptoms like breathlessness (dyspnea), fatigue, and brain fog (cognitive impairment) that persist or develop after SARS-CoV-2 infection [50]. For patients with severe COVID-19, rehabilitation programs are more complex, involving a comprehensive evaluation and medical care from a rehabilitation specialist, along with additional tests to identify related treatable post-COVID-19 conditions such as pulmonary fibrosis, hypothyroidism, glucose intolerance, anemia, cardiac arrhythmias, and cardiomyopathy [51]. Additionally, patients with moderate to severe cognitive impairments may undergo cerebral magnetic resonance imaging (MRI) with contrast or a computed tomography (CT) scan [51]. The rehabilitation program for severe cases includes activities such as exercises that gradually increase cardiovascular effort, as well as pulmonary rehabilitation, which involves the active cycle of breathing techniques, torso stretches, mobility exercises, and incentive spirometry. Moreover, the Borg Rating of Perceived Exertion and assessments like the Functional Independence Measure, 10 m walk test, 6 min walk test (6MWT), Timed Up and Go test, Jamar dynamometer for both hands, and Box and Blocks Test [51], along with the modified Medical Research Council (mMRC), dyspnea, fraction of inspired oxygen, and partial pressure of oxygen, are used to evaluate post-COVID-19 patients with severe cases following rehabilitation programs [52,53]. The study conducted by Shabat et al. included severe post-COVID-19 patients and reported that after 3 months of rehabilitation, all motor and cognitive parameters significantly improved, and most patients were able to wean off oxygen [51]. In some cases, the rehabilitation program for patients with severe conditions lasts between 4 and 8 weeks, and longer than 8 weeks when considering exercise capacity, peripheral muscle strength, and dyspnea [54].

Asimakos et al. conducted a non-randomized case–control study involving subjects with post-COVID-19 sequelae, where participants attended an 8-week, supervised rehabilitation program. Participation in rehabilitation was linked to significant clinical improvements in physical function, fatigue, activity, respiratory symptoms, and cognitive impairment [55]. Pulmonary rehabilitation (PR) lasts from 1 to 6 weeks with 2–5 sessions per week for acute COVID-19, and from 2 to 12 weeks with 2–5 sessions per week for chronic COVID-19. PR has led to a significant reduction in dyspnea among patients with COVID-19, whether mild or moderate/severe, compared to the control group [56]. Results from the three-week inpatient PR program, which included acute phase COVID-19 patients undergoing physical training, respiratory physiotherapy, and general physiotherapy, showed significant improvements in dyspnea, physical capacity, fatigue, and depression [42]. Pulmonary physiotherapy (PPT) is a comprehensive treatment approach designed to improve patients’ respiratory symptoms, teach effective coughing techniques, clear airways, reduce dyspnea, re-expand atelectatic lungs, provide short-term improvements in lung-thorax compliance, decrease disease-related complications, minimize disability, and ultimately enhance health-related quality of life. Therefore, PPT has been recommended as a strategy for hospitalized patients with various stages of COVID-19 pneumonia [57].

In some cases, rehabilitation for elderly individuals after acute COVID-19 can last longer than 4 weeks and involves combined approaches: exercise therapy, robotic gait training, occupational therapy, and massages [58]. Post-traumatic stress symptoms (PTSS) and breathlessness are well documented during the acute phase of COVID-19, as well as in post-COVID-19 syndrome (PCS), commonly known as Long COVID. The data collected from 154 participants experiencing persistent fatigue after acute COVID-19 infection, who enrolled in a 7-week rehabilitation program, indicate that PTSS may be a key treatment focus in multidisciplinary rehabilitation to help reduce fatigue during recovery from PCS [59].

Therefore, rehabilitation in severe cases takes a long time and provides many benefits, including improved health and functional outcomes, as well as support, especially for patients discharged from the Intensive Care Unit (ICU) [60].

### 4.9. Limitations and Further Directions

This study presents several limitations that should be taken into account when interpreting the results. First, its retrospective design and lack of a control group inherently limit the strength of the evidence. Although the observed improvements were statistically significant, these findings should be interpreted as associations rather than as proof of efficacy. Future prospective studies with appropriate control groups are needed to validate the effectiveness of the rehabilitation protocol. Second, the study relied exclusively on subjective assessment tools—namely, the Modified Borg Scales and the S5Q cooperation test—which, although validated and widely used in clinical rehabilitation, do not provide objective measures of physiological or functional capacity. The absence of pulmonary function tests, oxygen saturation monitoring, or standardized walking tests (e.g., the 6 min walk test) limits the ability to comprehensively assess cardiorespiratory performance and endurance. Future studies should combine both subjective and objective metrics to capture a more complete picture of recovery.

Third, while the S5Q scale is easy to administer and useful in clinical practice, it does not address psychosocial or motivational factors that may significantly influence rehabilitation outcomes. Its binary scoring system may also fail to detect subtle changes in responsiveness or engagement.

Fourth, the follow-up period was limited to a single rehabilitation cycle, with the second evaluation occurring only upon readmission. Therefore, the long-term sustainability of the observed improvements could not be assessed.

Fifth, several potentially confounding factors—such as comorbidities, ongoing medications, nutritional status, or social support—were not systematically analyzed; also, they are likely to influence recovery trajectories.

We also acknowledge the absence of objective physiological parameters such as heart rate and oxygen saturation, which are relevant indicators of cardiopulmonary recovery. Although these were monitored clinically, they were not systematically documented for research purposes and therefore could not be included in the analysis. This limitation reflects the evolving nature of clinical protocols during the early implementation phase of post-COVID-19 rehabilitation programs, rather than a methodological oversight.

Notably, at the time of data collection, the national rehabilitation protocol implemented in our center—developed in accordance with Ministry of Health guidelines—did not include standardized tools such as the 6MWT. As this study was based on retrospective analysis of routine clinical records, we were limited to the assessments officially adopted in the institutional protocol. We agree that future prospective studies should incorporate validated objective measures such as 6MWT and spirometry to strengthen the clinical and scientific value of rehabilitation research.

Furthermore, patients with oxygen saturation below 93% or those who experienced desaturation episodes were excluded from the active rehabilitation program and instead received only passive mobilization under nursing supervision. Once their clinical condition stabilized and their oxygen levels improved, these individuals became eligible for structured rehabilitation, but they were not included in the present cohort. Some patients with variable recovery trajectories did undergo spirometry after the acute phase, but were likewise excluded due to protocol-based criteria. These predefined exclusions, derived from national recommendations at the time, further limited the inclusion of objective cardiopulmonary data and restricted the study population to individuals with mild or moderate post-COVID-19 presentations.

In summary, future studies should adopt prospective and controlled designs with longer follow-up intervals, integrate standardized objective assessments (e.g., 6MWT, spirometry, oxygen saturation, heart rate), and include psychological, cognitive, and social dimensions. This comprehensive approach will help generate a more holistic understanding of rehabilitation outcomes and inform the development of individualized, evidence-based care strategies.

We acknowledge the importance of including objective functional measures such as the 6MWT in rehabilitation research. However, at the time of data collection, the national rehabilitation protocol implemented in our center—developed in accordance with the Ministry of Health guidelines—did not include the 6MWT as a standardized assessment tool for post-COVID-19 recovery. As our study was retrospective and based on routine clinical documentation, we were limited to the evaluation methods officially included in the protocol. We agree that future prospective studies should incorporate validated objective measures such as the 6MWT and spirometry to provide a more comprehensive understanding of recovery trajectories.

Another important limitation is the absence of objective physiological parameters, such as heart rate and oxygen saturation, which are relevant for assessing cardiopulmonary recovery. Although these measurements were routinely monitored in the clinical setting, they were not systematically recorded as part of the rehabilitation documentation and therefore could not be included in the retrospective dataset. This gap reflects limitations in protocol standardization during the early implementation of post-COVID-19 rehabilitation programs, rather than a methodological omission. Future prospective studies should ensure the consistent integration of such objective measures alongside patient-reported outcomes to strengthen the assessment of rehabilitation efficacy.

Another important limitation is the absence of objective physiological parameters such as heart rate and oxygen saturation, which are typically used to assess cardiopulmonary recovery. Although heart rate and blood pressure were monitored daily in all patients undergoing rehabilitation, their values did not show statistically significant variations and were not systematically recorded for research purposes. Moreover, patients with oxygen saturation below 93% or those who experienced desaturation episodes were excluded from the structured rehabilitation program, as they were classified in the severe phase of the disease. In these cases, only passive mobilization procedures were implemented during hospitalization, under the supervision of nursing staff. Once these patients progressed beyond the acute stage of COVID-19 and their oxygen saturation stabilized, they became eligible for inclusion in active rehabilitation programs. Some individuals with variable recovery trajectories underwent spirometry after testing negative and completing the febrile phase, but they were not included in the present cohort. These protocol-based exclusions—derived from national clinical recommendations at the time—limited the inclusion of objective cardiopulmonary measures and restricted the scope of this study to patients with mild-to-moderate presentations. Future prospective investigations should incorporate standardized collection of physiological data and objective functional parameters (e.g., spirometry, 6 min walk test, heart rate, and oxygen saturation) to strengthen the validity of rehabilitation outcome assessments.

## 5. Conclusions

This retrospective study shows statistically and clinically significant improvements in cooperation, effort tolerance, and dyspnea perception among patients with mild and moderate post-COVID-19. Across the entire cohort and in subgroup analyses by gender and age, cooperation scores consistently increased after recovery, while effort and dyspnea scores significantly decreased, indicating positive rehabilitative progress.

The magnitude of change was similar across men and women, as well as across different age groups, although there were slight trends toward greater improvement in male and younger patients. Notably, patients who started with higher levels of cooperation tended to report lower perceived effort, indicating that early behavioral engagement might positively influence recovery. Additionally, the very strong correlation between initial effort and dyspnea scores highlights the close connection between physical exertion and respiratory discomfort during the early post-acute phase.

These findings emphasize the importance of structured follow-up and rehabilitation for patients recovering from mild and moderate COVID-19, especially in improving effort adaptation and respiratory comfort. Although the data indicate a general trend of functional recovery, individual differences remain high, emphasizing the need for personalized therapeutic approaches.

Future research should examine the long-term stability of these improvements, the predictive power of baseline scores, and the effectiveness of targeted rehabilitation protocols customized for specific symptom clusters or functional deficits.

## Figures and Tables

**Table 1:** Cooperation, effort, and dyspnea scores before and after the recovery program in women and men.

Parameters		Mean ± SD	Median (IQR)	*p*-Value
Cooperation	Initial (*p* < 0.001 )	3.79 ± 0.85	4 (3–5)	<0.001
	After recovery (*p* < 0.001)	4.27 ± 0.71	4 (4–5)	<0.01
Effort	Initial (*p* < 0.001)	5.54 ± 1.35	6 (4–7)	<0.01
	After recovery (*p* < 0.001)	4.55 ± 1.18	4.5 (4–5)	<0.01
Dyspnea	Initial (*p* < 0.001)	5.62 ± 1.4	6 (4–7)	<0.01
	After recovery (*p* < 0.001)	4.57 ± 1.2	5 (4–5)	<0.01
Women’s cooperation scores	Initial (*p* < 0.001)	3.82 ± 0.86	4 (3–5)	<0.01
	After recovery (*p* < 0.001)	4.25 ± 0.79	4 (4–5)	<0.01
Women’s effort scores	Initial (*p* < 0.001)	5.4 ± 1.31	5 (4–6.5)	<0.01
	After recovery (*p* < 0.001)	4.45 ± 1.15	4 (3–5)	<0.01
Women’s dyspnea scores	Initial (*p* < 0.001)	5.48 ± 1.38	6 (4–7)	<0.01
	After recovery (*p* < 0.001)	4.47 ± 1.18	4 (3–5)	<0.01
Men’s cooperation scores	Initial (*p* < 0.001)	3.73 ± 0.82	3 (3–4)	<0.01
	After recovery (*p* < 0.001)	4.3 ± 0.58	4 (4–5)	<0.01
Men’s effort scores	Initial (*p* = 0.011)	5.76 ± 1.38	6 (5–7)	<0.01
	After recovery (*p* = 0.00)	4.7 ± 1.22	5 (4–6)	<0.01
Men’s dyspnea scores	Initial (*p* = 0.006)	5.83 ± 1.42	6 (5–7)	<0.01
	After recovery (*p* = 0.002)	4.71 ± 1.22	5 (4–6)	<0.01

**Table 2:** Cooperation, effort, and dyspnea scores before and after the recovery program by age.

Parameter		Mean ± SD	Median (IQR)	*p*-Value
Patients’ cooperation scores under 60 years old	Initial (*p* < 0.001)	4.07 ± 0.84	4 (3–5)	<0.01
	After recovery (*p* < 0.001)	4.49 ± 0.63	5 (4–5)	<0.001
Patients’ effort scores under 60 years old	Initial (*p* < 0.001)	5.14 ± 1.33	5 (4–6)	<0.01
	After recovery (*p* < 0.001)	4.14 ± 1.15	4 (3–5)	<0.01
Patients’ dyspnea scores under 60 years old	Initial (*p* < 0.001)	5.24 ± 1.44	5 (4–6.5)	<0.01
	After recovery (*p* < 0.001)	4.15 ± 1.18	4 (3–5)	<0.01
Patients’ cooperation scores ≥ 60 years	Initial (*p* < 0.001)	3.47 ± 0.74	3 (3–4)	<0.01
	After recovery (*p* < 0.001)	4.01 ± 0.73	4 (3–5)	<0.01
Patients’ effort scores ≥ 60 years	Initial (*p* < 0.001)	6 ± 1.23	6 (5–7)	<0.01
	After recovery (*p* < 0.001)	5.01 ± 1.05	5 (4–6)	<0.01
Patients’ dyspnea scores ≥ 60 years	Initial (*p* = 0.006 )	6.04 ± 1.23	6 (5–7)	<0.01
	After recovery (*p* = 0.002 )	5.04 ± 1.05	5 (4–6)	<0.01

**Table 3:** Cooperation, effort, and dyspnea scores before and after the recovery program by gender and Age.

Parameter		Mean ± SD	Median (IQR)	Mean Rank	*p*-Value
Cooperation Scores by Gender	Female (*p* < 0.001 )	0.42 ± 0.62	0 (0–1)	75.72	0.065
	Male (*p* < 0.001 )	0.57 ± 0.56	1 (0–1)	87.86	0.065
Effort Scores by Gender	Female (*p* < 0.001 )	−0.95 ± 1	−1 (−1–0)	81.78	0.639
	Male (*p* < 0.001 )	−1.06 ± 1	−1 (−1–0)	78.52	0.639
Dyspnea Scores by Gender	Female (*p* < 0.001 )	−1.01 ± 1.02	−1 (−1–0)	82.09	0.565
	Male (*p* < 0.001)	−1.11 ± 0.97	−1 (−2–0)	78.06	0.565
Cooperation Scores by Age	<60 years (*p* < 0.001 )	0.42 ± 0.56	0 (0–1)	76.34	0.220
	≥60 years (*p* < 0.001 )	0.54 ± 0.64	0 (0–1)	84.21	0.220
Effort Scores by Age	<60 years (*p* < 0.001 )	−1 ± 1.07	−1 (−1–0)	82.64	0.403
	≥60 years (*p* < 0.001 )	−0.97 ± 0.9	−1 (−1–−0.75)	76.97	0.403
Dyspnea Scores by Age	<60 years (*p* < 0.001)	−1.08 ± 1.08	−1 (−1.5–0)	80.84	0.793
	≥60 years (*p* < 0.001)	−1 ± 0.9	−1 (−1.25–0)	79.04	0.793

**Table 4:** Correlation between initial cooperation and effort scores.

Correlation	*p* *
Cooperation (*p* < 0.001 **) x Effort (*p* < 0.001 **)	<0.001, R = −0.571

* Spearman’s rho correlation coefficient, ** Shapiro–Wilk test.

**Table 5:** Correlation between initial effort and dyspnea scores.

Correlation	*p* *
Effort (*p* < 0.001 **) x Dyspnea (*p* < 0.001 **)	<0.001, R = 0.942

* Spearman’s rho correlation coefficient, ** Shapiro–Wilk test.

## Data Availability

The data presented in this study are available on request from the corresponding author. The data are not publicly available due to privacy and ethical restrictions.

## Abbreviations

The following abbreviations are used in this manuscript:

SARS-CoV-2	Severe Acute Respiratory Syndrome Coronavirus 2

## References

[1] Che Mohd Nassir C.M.N., Hashim S., Wong K.K., Abdul Halim S., Idris N.S., Jayabalan N., Guo D., Mustapha M. (2021). COVID-19 Infection and Circulating Microparticles—Reviewing Evidence as Microthrombogenic Risk Factor for Cerebral Small Vessel Disease. Mol. Neurobiol..

[2] Majumder J., Minko T. (2021). Recent Developments on Therapeutic and Diagnostic Approaches for COVID-19. AAPS J..

[3] Shan D., Wang C., Crawford T., Holland C. (2024). Association between COVID-19 Infection and New-Onset Dementia in Older Adults: A Systematic Review and Meta-Analysis. BMC Geriatr..

[4] Lui G., Guaraldi G. (2023). Drug Treatment of COVID-19 Infection. Curr. Opin. Pulm. Med..

[5] Parasher A. (2021). COVID-19: Current Understanding of Its Pathophysiology, Clinical Presentation and Treatment. Postgrad. Med. J..

[6] Ora J., Liguori C., Puxeddu E., Coppola A., Matino M., Pierantozzi M., Mercuri N.B., Rogliani P. (2020). Dyspnea Perception and Neurological Symptoms in Non-Severe COVID-19 Patients. Neurol. Sci..

[7] Ries A.L. (2005). Minimally Clinically Important Difference for the UCSD Shortness of Breath Questionnaire, Borg Scale, and Visual Analog Scale. COPD J. Chronic Obstr. Pulm. Dis..

[8] Tache-Codreanu D.-L., Bobocea L., David I., Burcea C.-C., Sporea C. (2024). The Role of the Six-Minute Walk Test in the Functional Evaluation of the Efficacy of Rehabilitation Programs After COVID-19. Life.

[9] Jafarnezhadgero A.A., Noroozi R., Fakhri E., Granacher U., Oliveira A.S. (2022). The Impact of COVID-19 and Muscle Fatigue on Cardiorespiratory Fitness and Running Kinetics in Female Recreational Runners. Front. Physiol..

[10] Scherr J., Wolfarth B., Christle J.W., Pressler A., Wagenpfeil S., Halle M. (2013). Associations between Borg’s Rating of Perceived Exertion and Physiological Measures of Exercise Intensity. Eur. J. Appl. Physiol..

[11] Wakefield J.C. (2007). The Concept of Mental Disorder: Diagnostic Implications of the Harmful Dysfunction Analysis. World Psychiatry.

[12] Soriano J.B., Murthy S., Marshall J.C., Relan P., Diaz J.V. (2022). A Clinical Case Definition of Post-COVID-19 Condition by a Delphi Consensus. Lancet Infect. Dis..

[13] Shaikh S., Siddiqi Z., Ukachukwu C., Mehkari Z., Khan S., Pamurthy K., Jahan F., Brown A. (2023). COVID-19: Post-Recovery Manifestations. Cureus.

[14] Jimeno-Almazán A., Buendía-Romero Á., Martínez-Cava A., Franco-López F., Sánchez-Alcaraz B.J., Courel-Ibáñez J., Pallarés J.G. (2023). Effects of a Concurrent Training, Respiratory Muscle Exercise, and Self-Management Recommendations on Recovery from Post-COVID-19 Conditions: The RECOVE Trial. J. Appl. Physiol..

[15] Cascella M., Rajnik M., Aleem A., Dulebohn S.C., Di Napoli R. (2025). Features, Evaluation, and Treatment of Coronavirus (COVID-19).

[16] Williams N. (2017). The Borg Rating of Perceived Exertion (RPE) Scale. Occup. Med..

[17] Abu-Odah H., Liu X.-L., Wang T., Zhao I.Y., Yorke J., Tan J.-Y.B., Molassiotis A. (2025). Modified Borg Scale (MBorg), the Numerical Rating Scale (NRS), and the Dyspnea-12 Scale (D-12): Cross-Scale Comparison Assessing the Development of Dyspnea in Early-Stage Lung Cancer Patients. Support. Care Cancer.

[18] Jang M.H., Shin M.-J., Shin Y.B. (2019). Pulmonary and Physical Rehabilitation in Critically Ill Patients. Acute Crit. care.

[19] Mao L., Luo L., Wang D., Yu Y., Dong S., Zhang P., Sun Y., Chen Z. (2021). Early Rehabilitation after Lung Transplantation with Extracorporeal Membrane Oxygenation (ECMO) of COVID-19 Patient: A Case Report. Ann. Transl. Med..

[20] Shapiro S.S., Wilk M.B., Chen H.J. (1968). A Comparative Study of Various Tests for Normality. J. Am. Stat. Assoc..

[21] Rey D., Neuhäuser M. (2011). Wilcoxon-Signed-Rank Test. International Encyclopedia of Statistical Science.

[22] Wall Emerson R. (2023). Mann-Whitney U Test and t-Test. J. Vis. Impair. Blind..

[23] Beyer S., Haufe S., Dirks M., Scharbau M., Lampe V., Dopfer-Jablonka A., Tegtbur U., Pink I., Drick N., Kerling A. (2023). Post-COVID-19 Syndrome: Physical Capacity, Fatigue and Quality of Life. PLoS ONE.

[24] Zheng C., Chen X.-K., Sit C.H., Liang X., Li M.-H., Ma A.C., Wong S.H. (2023). Effect of Physical Exercise-Based Rehabilitation on Long COVID: A Systematic Review and Meta-Analysis. Med. Sci. Sports Exerc..

[25] Jones E.A.K., Mitra A.K., Bhuiyan A.R. (2021). Impact of COVID-19 on Mental Health in Adolescents: A Systematic Review. Int. J. Environ. Res. Public Health.

[26] Alosaimi N., Sherar L.B., Griffiths P., Pearson N. (2023). Clustering of Diet, Physical Activity and Sedentary Behaviour and Related Physical and Mental Health Outcomes: A Systematic Review. BMC Public Health.

[27] Hawes M.T., Szenczy A.K., Klein D.N., Hajcak G., Nelson B.D. (2022). Increases in Depression and Anxiety Symptoms in Adolescents and Young Adults during the COVID-19 Pandemic. Psychol. Med..

[28] Özgüç S., Kaplan Serin E., Tanriverdi D. (2024). Death Anxiety Associated with Coronavirus (COVID-19) Disease: A Systematic Review and Meta-Analysis. OMEGA J. Death Dying.

[29] Alacevich C., Thalmann I., Nicodemo C., de Lusignan S., Petrou S. (2023). Depression and Anxiety during and after Episodes of COVID-19 in the Community. Sci. Rep..

[30] Kızılkaya S., Çağatay A. (2023). Detrás Del Backstage de La Pandemia de COVID-19: Ansiedad y Trabajadores de La Salud. Cir. Cir..

[31] Rodriguez-Ayllon M., Cadenas-Sánchez C., Estévez-López F., Muñoz N.E., Mora-Gonzalez J., Migueles J.H., Molina-García P., Henriksson H., Mena-Molina A., Martínez-Vizcaíno V. (2019). Role of Physical Activity and Sedentary Behavior in the Mental Health of Preschoolers, Children and Adolescents: A Systematic Review and Meta-Analysis. Sports Med..

[32] Herbert C. (2022). Enhancing Mental Health, Well-Being and Active Lifestyles of University Students by Means of Physical Activity and Exercise Research Programs. Front. Public Health.

[33] da Silva L.A., Tortelli L., Motta J., Menguer L., Mariano S., Tasca G., de Bem Silveira G., Pinho R.A., Silveira P.C.L. (2019). Effects of Aquatic Exercise on Mental Health, Functional Autonomy and Oxidative Stress in Depressed Elderly Individuals: A Randomized Clinical Trial. Clinics.

[34] Cunningham C., O’ Sullivan R., Caserotti P., Tully M.A. (2020). Consequences of Physical Inactivity in Older Adults: A Systematic Review of Reviews and Meta-analyses. Scand. J. Med. Sci. Sports.

[35] del Corral T., Fabero-Garrido R., Plaza-Manzano G., Fernández-de-las-Peñas C., Navarro-Santana M., López-de-Uralde-Villanueva I. (2023). Home-Based Respiratory Muscle Training on Quality of Life and Exercise Tolerance in Long-Term Post-COVID-19: Randomized Controlled Trial. Ann. Phys. Rehabil. Med..

[36] Jack K., McLean S.M., Moffett J.K., Gardiner E. (2010). Barriers to Treatment Adherence in Physiotherapy Outpatient Clinics: A Systematic Review. Man. Ther..

[37] Oliveira M.R., Hoffman M., Jones A.W., Holland A.E., Borghi-Silva A. (2024). Effect of Pulmonary Rehabilitation on Exercise Capacity, Dyspnea, Fatigue, and Peripheral Muscle Strength in Patients with Post-COVID-19 Syndrome: A Systematic Review and Meta-Analysis. Arch. Phys. Med. Rehabil..

[38] McNarry M.A., Berg R.M.G., Shelley J., Hudson J., Saynor Z.L., Duckers J., Lewis K., Davies G.A., Mackintosh K.A. (2022). Inspiratory Muscle Training Enhances Recovery Post-COVID-19: A Randomised Controlled Trial. Eur. Respir. J..

[39] Sánchez-Milá Z., Abuín-Porras V., Romero-Morales C., Almazán-Polo J., Velázquez Saornil J. (2023). Effectiveness of a Respiratory Rehabilitation Program Including an Inspiration Training Device versus Traditional Respiratory Rehabilitation: A Randomized Controlled Trial. PeerJ.

[40] Frazão M., da Cruz Santos A., Silva P.E., de Assis Pereira Cacau L., Petrucci T.R., Assis M.C., de Almeida Leal R., Brasileiro E., de Moraes Forjaz C.L., do Socorro Brasileiro-Santos M. (2023). Impaired Neuromuscular Efficiency and Symptom-Limited Aerobic Exercise Capacity 4 Weeks After Recovery From COVID-19 Appear to Be Associated With Disease Severity at Onset. Phys. Ther..

[41] Li H., Chen J., Yu Y., Mao L., Luo L., Zou L., Zhang T., Yang J., Chen Z. (2022). Early Physical Therapy for a Patient Affected by Coronavirus Disease 2019 (COVID-19) on Awake Veno-Venous Extracorporeal Membrane Oxygenation: A Case Report. Ann. Transl. Med..

[42] Hayden M.C., Limbach M., Schuler M., Merkl S., Schwarzl G., Jakab K., Nowak D., Schultz K. (2021). Effectiveness of a Three-Week Inpatient Pulmonary Rehabilitation Program for Patients after COVID-19: A Prospective Observational Study. Int. J. Environ. Res. Public Health.

[43] Fugazzaro S., Contri A., Esseroukh O., Kaleci S., Croci S., Massari M., Facciolongo N.C., Besutti G., Iori M., Salvarani C. (2022). Rehabilitation Interventions for Post-Acute COVID-19 Syndrome: A Systematic Review. Int. J. Environ. Res. Public Health.

[44] Filipović T., Gajić I., Gimigliano F., Backović A., Hrković M., Nikolić D., Filipović A. (2023). The Role of Acute Rehabilitation in COVID-19 Patients. Eur. J. Phys. Rehabil. Med..

[45] da Vieira A.G.S., Pinto A.C.P.N., Garcia B.M.S.P., Eid R.A.C., Mól C.G., Nawa R.K. (2022). Telerehabilitation Improves Physical Function and Reduces Dyspnoea in People with COVID-19 and Post-COVID-19 Conditions: A Systematic Review. J. Physiother..

[46] Pouliopoulou D.V., Macdermid J.C., Saunders E., Peters S., Brunton L., Miller E., Quinn K.L., Pereira T.V., Bobos P. (2023). Rehabilitation Interventions for Physical Capacity and Quality of Life in Adults with Post–COVID-19 Condition. JAMA Netw. Open.

[47] Elyazed T.I.A., Alsharawy L.A., Salem S.E., Helmy N.A., El-Hakim A.A.E.-M.A. (2024). Effect of Home-Based Pulmonary Rehabilitation on Exercise Capacity in Post COVID-19 Patients: A Randomized Controlled Trail. J. Neuroeng. Rehabil..

[48] Pleguezuelos E., Sánchez-Nuño S., Del Carmen A., Serra-Payá N., Moreno E., Molina-Raya L., Robleda G., Benet M., Santos-Ruiz S., Garrido A.B. (2023). Effect of Different Types of Supervised Exercise Programs on Cardiorespiratory and Muscular Fitness, Pain, Fatigue, Mental Health and Inflammatory and Oxidative Stress Biomarkers in Older Patients with Post-COVID-19 Sequelae “EJerSA-COVID-19”: A randomized controlled trial. BMC Geriatr..

[49] Olezene C.S., Hansen E., Steere H.K., Giacino J.T., Polich G.R., Borg-Stein J., Zafonte R.D., Schneider J.C. (2021). Functional Outcomes in the Inpatient Rehabilitation Setting Following Severe COVID-19 Infection. PLoS ONE.

[50] Nalbandian A., Sehgal K., Gupta A., Madhavan M.V., McGroder C., Stevens J.S., Cook J.R., Nordvig A.S., Shalev D., Sehrawat T.S. (2021). Post-Acute COVID-19 Syndrome. Nat. Med..

[51] Shabat S., Marmor A., Shiri S., Tsenter J., Meiner Z., Schwartz I. (2023). Correlations between Disease Severity and Rehabilitation Outcomes in Patients Recovering from Covid-19 Infection. J. Rehabil. Med..

[52] Liu S., Zhan C., Ma Y., Guo C., Chen W., Fang X., Fang L. (2021). Effect of Qigong Exercise and Acupressure Rehabilitation Program on Pulmonary Function and Respiratory Symptoms in Patients Hospitalized with Severe COVID-19: A Randomized Controlled Trial. Integr. Med. Res..

[53] Curci C., Pisano F., Bonacci E., Camozzi D.M., Ceravolo C., Bergonzi R., De Franceschi S., Moro P., Guarnieri R., Ferrillo M. (2020). Early Rehabilitation in Post-Acute COVID-19 Patients: Data from an Italian COVID-19 Rehabilitation Unit and Proposal of a Treatment Protocol. Eur. J. Phys. Rehabil. Med..

[54] Singh S.J., Baldwin M.M., Daynes E., Evans R.A., Greening N.J., Jenkins R.G., Lone N.I., McAuley H., Mehta P., Newman J. (2023). Respiratory Sequelae of COVID-19: Pulmonary and Extrapulmonary Origins, and Approaches to Clinical Care and Rehabilitation. Lancet Respir. Med..

[55] Asimakos A., Spetsioti S., Mentzelopoulos S., Vogiatzis I., Vassiliou A.G., Gounopoulos P., Antonoglou A., Spaggoulakis D., Pappa S., Zakynthinos S. (2024). Rehabilitation Is Associated with Improvements in Post–COVID-19 Sequelae. Respir. Care.

[56] Ahmed I., Mustafaoglu R., Yeldan I., Yasaci Z., Erhan B. (2022). Effect of Pulmonary Rehabilitation Approaches on Dyspnea, Exercise Capacity, Fatigue, Lung Functions, and Quality of Life in Patients With COVID-19: A Systematic Review and Meta-Analysis. Arch. Phys. Med. Rehabil..

[57] Javaherian M., Shadmehr A., Keshtkar A., Beigmohammadi M.T., Dabbaghipour N., Syed A., Attarbashi Moghadam B. (2023). Safety and Efficacy of Pulmonary Physiotherapy in Hospitalized Patients with Severe COVID-19 Pneumonia (PPTCOVID Study): A Prospective, Randomised, Single-Blind, Controlled Trial. PLoS ONE.

[58] Cevei M., Onofrei R.R., Gherle A., Gug C., Stoicanescu D. (2022). Rehabilitation of Post-COVID-19 Musculoskeletal Sequelae in Geriatric Patients: A Case Series Study. Int. J. Environ. Res. Public Health.

[59] Harenwall S., Heywood-Everett S., Henderson R., Smith J., McEnery R., Bland A.R. (2022). The Interactive Effects of Post-Traumatic Stress Symptoms and Breathlessness on Fatigue Severity in Post-COVID-19 Syndrome. J. Clin. Med..

[60] Waluyo Y., Artika S.R., Wahyuni I.N., Valen S.D., Sam N. (2021). Optimizing Early Rehabilitation Intervention: Insights from Different Outcomes in 2 Patients with Severe COVID-19. Am. J. Case Rep..

